# Treatment of Burns—Lessons from the “Richmond” Fire*Read before the Buffalo Medical Union, May, 1887.

**Published:** 1887-09

**Authors:** George E. Fell

**Affiliations:** Professor of Physiology and Microscopy, Niagara University


### * *TREATMENT OF BURNS—LESSONS FROM THE "RICHMOND" FIRE.*

By GEORGE E. FELL, M. D., F. R. M. S.,

Professor of Physiology and Microscopy, Niagara University.

To many physicians the experiences produced through a disaster so terrible as the burning of a hotel full of valuable lives may never come. No one, however, can feel assured that it will not be his duty to render aid in such an emergency; hence all should be prepared for such service. In connection with the duties of the surgeon, I have divided my remarks into the following heads:

1st.—The immediate duty to the suffering patients, temporary dressings and relief of pain.

2d.—The removal of the injured to hospitals or homes.

3d.—Dressings of wounds of a more permanent character.

4th.—Subsequent treatment with a consideration of shock.

5th.—Prognosis in burns.

*Under the head of immediate duty to the patient.*—At one of the hospitals on the morning after the fire, I found several of the sufferers with their wounds dressed with ordinary cotton, apparently placed dry upon the wounds. The exuded serum had caused it to adhere closely, so that in the attempt at removal difficulty was experienced and severe pain produced before the permanent dressing could be applied. Had the parts or the cotton been saturated with oil, it would have been much better. Drug stores were in close proximity to the scene of the disaster, from which such material might have been procured. Sur-

---

* Read before the Buffalo Medical Union, May, 1887.

geon's lint, which may be torn in strips or used in large pieces, may be saturated with the oil and lightly bandaged on the wound. Better than the dry cotton application would be a simple bandage saturated with oil.

*Relief of pain.*—Morphia and atropia, hypodermically, it appears to me most generally indicated, one-eighth to one-half grain, according to extent of the injuries.

Regarding temporary dressings, sweet or linseed oil is mentioned as being easily procured.

*The removal of the injured to hospitals and homes.*—Here the duty of the physician comes in with weighty responsibility. The condition of the patient, however serious, generally requires the prompt removal to the hospital or home. As the ambulances are seldom made to carry more than two patients, and often but one comfortably, we have a difficulty meeting us at the threshold of this inquiry. Then, again, when large numbers are to be quickly transported, as in the case of the late disaster, and the emergency hospitals are full, the distance of the large hospitals,—the General and Sisters'—so far as Buffalo is concerned, gives another objection to the transportation by small ambulance. Had several of Mr. C. W. Miller's large covered wagons or large express wagons been called into the service, much better and quicker work might have been done. The placing of straw and mattresses in these wagons, with the use of a good number of stretchers, would make them fully as serviceable as the ordinary ambulance. A little forethought, or the establishment of an *emergency* ambulance system in different cities, would prevent the packing of suffering human beings into two or three small ambulances, as was noted at the Richmond fire.

In a disaster like the Richmond, the question arises as to the best time to send patients to their homes in the city or at a distance sometimes of hundreds of miles.

It would be well if all patients living in the city or town where the disaster occurs, after having their wounds dressed, could be immediately taken with care to their homes. This rule would be applicable to those going to hospitals in the city for the following reasons: The effect of shock in burns is frequently not seen in from one to twenty-four hours to forty-eight hours, or even later; during this time patients can be moved with the least danger. However, when a long journey is to be undertaken, it appears that the effect of shock should be determined first, and after that, if the journey must be undertaken, let it be done with the greatest precautions, with the patient under the immediate care of a physician. There are several instances recalled from the Richmond disaster where patients seriously injured and removed to their homes at a distance had great difficulty in recovery. It is reasonable to think that a long railway journey would at the very least be injurious to the patient under such conditions.

*Dressing of wounds of a more permanent character.*—In a paper read by Dr. Lynde at the last meeting of this society, we found that they were almost as numerous as the nostrums for the cure of rheumatism. We trust that generally they may be more serviceable. In the talk of the laity there are mentioned many wonderful effective cures for burns, as "Dally's" salve, "Chapsega" oil, etc., etc. In examining their merits we find it exists in the properties they have of preventing access of air to the wound.

While it may be in the eyes of the medical profession a prevalent impression that nature does all in the process of repair of tissues, it is undoubtedly true that certain agents, through their effectiveness in producing a covering or condition similating the natural, may, therefore, enable the process of repair to be more expeditiously carried on. I will briefly refer to the dressings used in the cases about which this paper centers, not because they may have any special virtues that other dressings have not, but rather that time will not permit another course.

The first dressing used on one of my patients was the Carron oil without any addition. This was obtained at the druggist's, and with saturated surgeon's lint was applied to his hands. Afterwards non-absorbing cotton was used for several times with the oil, and sufficiently long to demonstrate its utter worthlessness for this purpose. A dressing which becomes attached to the tissue is, in my opinion, objectionable.

Among the other dressings used may be mentioned collodium, which is only applicable to small surfaces, and never should be applied to large ones; oxide of zinc and Carron oil, objectionable both on account of its appearance and difficulty of removal; iodoform powder and lanolin, or vaselin, and iodoform powder and boracic acid, the latter objectionable through the production of pain; iodoform powder and starch in various proportions and Carron oil, and naphthaline. Of the dressings, I will say that cotton was early discarded even as an outside dressing. Finally, the dressing which gave the greatest satisfaction was that made of about equal parts of iodoform powder and corn starch with Carron oil, and used as follows: The dry powder was dusted upon the wounded surfaces with a duster—a "baby's puff ball," those used in the nursery being found to be the best. With this all portions of the wound could be thoroughly sprinkled. The Carron oil was then poured over the powder to cover all parts of the wounds; on this a common roller bandage, thoroughly saturated with the oil, was carefully but loosely wound, and the whole covered with layers of oiled silk, sufficient to prevent the escape of the oil.

A dressing of mutton tallow and a zinc oxyde was used, in which the fibres are removed from the tallow, which is afterwards kneaded in a mortar until soft and pliable; it is then cut into strips and applied to the wound and secured with bandages. This dressing was not found as convenient as the one previously mentioned.

The carbolized cosmoline dressing recommended and used in the University of Pennsylvania was not tried simply because the iodoform starch Carron oil mixture was found quite satisfactory.

When the Carron oil alone was used, within a short time the odor of putrefaction would make its appearance; when the iodoform was added to it as described, this unpleasant effect was prevented. The dressing had to be changed less frequently, and the surgeon had the satisfaction of knowing he was placing the wound under the best influence, not only so far as a soothing application affects the case, but to the believer in septic infection he had provided an additional safeguard in the repair of the wound.

Right here I may say I cannot see any more favorable condition under which a burn can be placed than that presented by this combination. We have the air excluded, a dressing that can be easily removed, and an antiseptic influence as good as any, and without many of the objections which others offer.

Naphthaline is a dressing which was recommended by the consultants in the case of some patients under my observation. Regarding its value, I find it highly commended.

*Naphthaline.*—It is said to be insoluble in water and the animal juices. In wounds it is to be used as a dry powder dressing, being dusted, in fine powder, on the surface of the wound or packed in quantities without limit into wound cavities. Pilcher also states that its antiseptic qualities are inferior to carbolic acid or iodoform, yet in addition to the general advantages that no general toxic effect ever follows its use, its freedom from toxic qualities commends it as a substitute for iodoform, etc.

It is also stated that a pure article of naphthaline only should be used; that an impure article is likely to cause pain and irritation of the wound, and that no such effects attend the use of pure naphthaline. I used Merk's preparation in the cases under my care.

According to this article, a better article than naphthaline could not possibly be used for a wounded surface. The use of it demonstrated to my satisfaction that it is not a fit substance to be used generally for burns as compared with iodoform and the Carron oil dressing. In the case of Mr. M., pain was experienced, as also in the case of Mrs. M., so that the dressings had to be changed with the happy result of relief when the iodoform and Carron oil was reapplied. As to the odor of the Carron oil and iodoform, I will only say that when the olfactory bulbs of a surgeon becomes so seriously affected that he cannot stand this slightly unpleasant odor for the benefit of his patient, who usually, if seriously injured, does not object to it, he had better take up the occupation of a horticulturist as more properly within his (nasal) sphere.

*Subsequent treatment with a consideration of shock.*—Shock in burns is the most serious factor. The extent of surface burned is infrequently an index of the extent of shock. The mental conditions associated with the disaster, the age and condition of the patient, must on this account be important factors in the production of this remarkable condition of the system. As the various functions of the body are intimately associated with the nerve centres and controlled through the efferent influences originated in or through these centres in the brain, it is quite probable that the condition termed shock is principally the effect of a disturbance of the centres of various functions in the brain.

The influence of a nerve centre on the functions of many portions of the body is indicated in the simple experiment of performing artificial respiration upon the lower animals. When the oxygen is withheld from the circulation, we have the attempts at respiration produced by the want of oxygen in the blood circulating in the medulla; the renewal of this want reproduces a condition similating the normal, and further respiratory efforts are not made.

When we consider the great number of centres of important physiological functions in the medulla alone, we may judge in a manner of the general effect of disturbing the normal condition of these centres by marked afferent influence. When the pneumogastrics are cut in the neck and central end stimulated, the hepatic functions are brought into action, it is supposed, through the sympathetic system, including its action in affecting the vasa motor system. In case of shock we have not only a decided interference with the centres of the brain interfering with the functional reflexes, but also have through the same influence a disarrangement of the vessels and organs of the system.

Moullin defines shock as "an example of reflex paralysis, in the strictest and narrowest sense of the term—a reflex inhibition, probably in the majority of cases generally affecting all of the nervous system," and not limited to the heart and vessels only. This undoubtedly is true, as we find produced through shock not only serious affection of the intestinal tract, but marked interference with the functions of the kidneys and liver, and other organs.

The effects of shock, usually at the best, passes away gradually. That the results in many cases is wonderfully modified by treatment was evidenced in the case of a Mrs. M., one of my patients. In the case of her hushand, who was also injured, it was weeks after the fire before it could be said he was out ot danger from shock; the degree of injury to the intestinal tract in this case being much less than in his wife's, but still evidently the result of the sadly peculiar conditions surrounding him, in the death of a wife and daughter, after a terrible physical and mental strain to which he had been subjected.

Case of a lady sufferer under my charge: I was called to attend Mrs. M., Sunday afternoon, March 20th, and was given immediate control of the patient by her father, a physician also; the case was to be carried along in consultation with other physicians interested in the case.

When first examined, the patient had a pulse of 150°, weak and threadlike; in fact, a pulse indicative of the most serious condition. On dressing the burns I found them more extensive than I expected. Both arms to the shoulders were badly burned, the fingers of the right hand, having been protected by a muff, were uninjured; both breasts had bad burns upon them; the neck was badly burned in front; the face, from the eyes to the throat, and on the upper portion of the back, existed two badly burned surfaces. Upon the arms in several places burns of the third degree were found near the elbows; with these exceptions the burns would be classed as of the second degree.

In the case of her husband, Mr. M., burns were found mostly of the second degree upon the hands and face, or including the entire surface of face and hands; on some portions of the hands the dermis was nearly burned away. Regarding the dear little girl, their daughter, whose life was sacrificed, I will say nothing further than that she was terribly burned, notwithstanding the reports to the contrary.

The therapeutical measures resorted to in these cases consisted of stimulants, heart tonics, combined with assimilable food and nourishing enemata. In addition, at my suggestion, the inhalation of pure oxygen was used, and with encouraging results. As a stimulant to the nerve centres, and from a physiological point of view, it was indicated. An agent used, but not frequently, is glonoin (nitro-glycerine); its efficacy in heart failure has been lauded by a Chicago physician. The ordinary dose is one drop of a one per cent. solution. In the case of Mrs. M., as a dernier resort gtt. I. of a two per cent. solution was used every three or four hours, as indicated. I can speak of this agent in the highest terms. It was used after all other forms of support appeared to fail, and the results were noted usually within a short time of administration. The results in the case of Mrs. M. seem to indicate the value of the means adapted, as from a weak and compressible pulse of 150 Sunday P. M., it was gradually reduced to 140, 135, 120, and on the Wednesday morning following to 108, with the respirations keeping pace. Owing to giving out of the stomach and lower bowels, so that nourishment could not be retained, with albuminuria setting in, the result could only go on to a fatal termination.

The case of Mr. M. presented some features of special interest in the long-continued depression of the system, which called for the most careful treatment. For the first four days the pulse was 120. It run up to 126 at one time; was gradually brought to 114 and 110, where it remained without much variation for another week or two, then down to 108. Stimulants, with ammonia and heart tonics, and most careful handling of the stomach, brought this case through successfully.

*Pronogsis in burns.*—If any disaster evidenced the importance of conservatism in the pronogsis in burns, this of the Richmond has demonstrated the necessity for it. In the case of both Mrs. M. and her little daughter, the reports at first were quite encouraging. From the newspapers I quote, March 18th: "Mrs. M., though extensively burned, is not apparently in danger." (*Express*, March 18th.) That the little girl would probably recover and that Mr. M. was but "slightly burned." When it is considered that the one who was slightly burned, two weeks after was not by any means considered out of danger, and that the two who were thought would recover, passed away, one two days and the other seven days after the disaster. Without calling into question any other cases, it will be seen that we have a lesson to learn right here. Without considering the fatality of shock, we have results sometimes arising from burns, as the ulceration of the duodenum and effects upon the abdominal viscera generally, which calls for at least a guarded prognosis on the part of the physician who values his reputation.

